# Efficient Embryogenic Callus Induction and *Agrobacterium*-Mediated Transformation of the Elite Foxtail Millet (*Setaria italica* L.) Variety Jingu51

**DOI:** 10.3390/plants15142159

**Published:** 2026-07-13

**Authors:** Huan-Huan Zhang, Li-Jiao Dai, Nan Xiao, Ying Zhou, Xin Hu, Chun-Hui Zhang, Ying-Ying Jia, Min Li, Yao-Shan Hao, Shen-Jie Wu

**Affiliations:** 1 Shanxi Hou Ji Laboratory, College of Life Science, Shanxi Agricultural University, Taiyuan 030031, China; zhang_hh@sxau.edu.cn (H.-H.Z.);; 2 College of Agriculture, Shanxi Agricultural University, Taiyuan 030031, China; 3 Key Laboratory of Minor Crop Germplasm Innovation and Molecular Breeding (Co-Construction by Ministry and Province), Ministry of Agriculture and Rural Affairs, Taiyuan 030031, China; 4 College of Grassland Science, Shanxi Agricultural University, Jinzhong 030801, China

**Keywords:** foxtail millet, embryogenic callus induction, copper sulfate, Agrobacterium-mediated transformation

## Abstract

Foxtail millet (*Setaria italica* L.) is an important food crop worldwide and serves as a model for studying C4 photosynthesis and drought resistance. However, its application in functional genomics and molecular breeding has been severely constrained by the absence of a reliable genetic transformation system. In this study, 66 varieties were systematically evaluated for embryogenic callus induction capacity, with rates ranging from 0 to 70.22%. Among these, five varieties exhibited high embryogenic potential (>50%). Jingu51, an elite cultivated variety in northern China, was selected as the transformation recipient due to its rapid embryogenic response and high induction frequency. Optimization of culture conditions revealed that Murashige & Skoog (MS) medium supplemented with 30 g·L^−1^ sucrose, 3 g·L^−1^ phytagel, 1 mg·L^−1^ CuSO_4_, and 1 mg·L^−1^ 2,4-dichlorophenoxyacetic acid combined with 0.5 mg·L^−1^ 6-benzylamino purine was optimal for embryogenic callus induction. Key parameters of Agrobacterium-mediated transformation, including 100 μM acetosyringone concentration, callus pretreatment (heat shock, followed by ice bath), bacterial density (OD_600_ = 0.3), and cocultivation temperature (19 °C), were optimized. Under these conditions, a robust genetic transformation system was successfully established, yielding an average efficiency of 9.25% by PCR across multiple expression vectors; half transgenic event exhibited single copy. Notably, the genetic transformation system of Jingu51, an elite variety of foxtail millet, provided a robust platform for functional genomics research and accelerated molecular breeding efforts in this important crop.

## 1. Introduction

Foxtail millet (*Setaria italica* L.) is an ancient crop originating from China that was domesticated and cultivated approximately 8700 years ago [1]. Due to its exceptional drought tolerance and adaptability to nutrient-poor soils, foxtail millet thrives in the arid and semi-arid environments of northern China. Prior to the 1980s, it served as one of the staple food crops in this region and remains one of the most widely cultivated minor cereal crops. Foxtail millet is characterized by high nutritional value and notable medicinal and health-promoting properties. It supports digestive health, exhibits hypolipidemic activity, possesses a low glycemic index, and demonstrates anti-inflammatory effects [2,3]. In recent years, foxtail millet has been increasingly recognized as an ideal C4 model system owing to its short life cycle, small genome size, and genetic tractability [4]. Recent advances in hybrid breeding technologies have further enhanced its yield, quality and economic value. However, compared with research on model plants such as *Arabidopsis thaliana* and rice (*Oryza sativa*), fundamental and biotechnological research on foxtail millet remains relatively limited [4,5]. With the emergence of modern breeding tools, particularly gene editing technologies, new opportunities have arisen for the genetic improvement of minor cereals such as foxtail millet [6].

Research on the genetic transformation of foxtail millet has advanced considerably in recent years. Wang et al. established an *Agrobacterium*-mediated transformation system for foxtail millet using immature inflorescences of Jigu11 as explants, achieving transformation efficiency of 5.5% [7]. However, immature inflorescences were limited only in special season and difficult to operate. Ceasar et al. established an *Agrobacterium*-mediated transformation system through shoot tip infection using genotype, achieving a transformation frequency of approximately 9% [8]. Shoot apex dissection is technically demanding and prone to contamination, and the regeneration pathway via organogenesis produces more chimeric plants than embryogenesis-based approaches. Mature embryos offer year-round availability, simple preparation, and high embryogenic callus induction consistency. These advantages make mature embryos the preferred explant type for developing a broadly applicable transformation system in foxtail millet. Yang et al. further established a transformation system using the mini-foxtail millet variety *xiaomi* [4]. While this system serves as an excellent tool for basic functional genomics, *xiaomi* is not an agronomically elite variety, and the transformation protocol has not been demonstrated to be transferable to commercial cultivars. At the same time, Santos et al. and Sood et al. developed more efficient transformation systems using mature seeds as explants, with frequencies reaching 19.2% and 27%, respectively [9,10]. However, these transformation protocols remain accessible to only a few laboratories (Yugu1 and IC-403579/IC-480117), with limited agronomic relevance. The lack of a reliable transformation system for agronomically important cultivars therefore remains a critical bottleneck impeding the application of gene editing technologies in foxtail millet improvement.

In this study, we systematically screened 66 foxtail millet varieties to identify genotypes with high embryogenic potential. Jingu51, which exhibited superior embryogenic capacity, was selected as the experimental material. We subsequently optimized the composition of the callus induction medium to maximize embryogenic callus proliferation and established a reliable and stable genetic transformation system for foxtail millet, providing a valuable platform for functional genomics and molecular breeding in this crop.

## 2. Results

### 2.1. Screening of High-Frequency Embryogenic Varieties in Foxtail Millet

**Figure 1:**
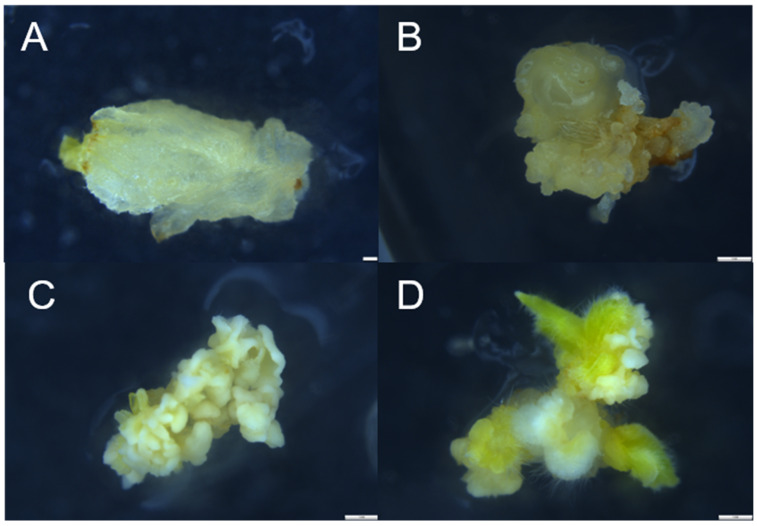
Morphological stages of callus development in foxtail millet. (**A**) Primary callus. (**B**) Dedifferentiated callus. (**C**) Embryogenic callus. (**D**) Green shoot bud formation on the surface of an embryonic callus. Scale bar = 1 mm.

Genetic transformation relies heavily on the dedifferentiation and redifferentiation potential of explants. In this study, we observed that foxtail millet mature embryos produce superior embryogenic calli, identified by their characteristic yellow or white color, compact texture, and lamellar morphology. These features allow for easy identification through visual inspection. We demonstrate that this embryogenic state is a prerequisite for shoot bud regeneration, which is absent in non-embryogenic calli (Figure 1).

**Figure 2:**
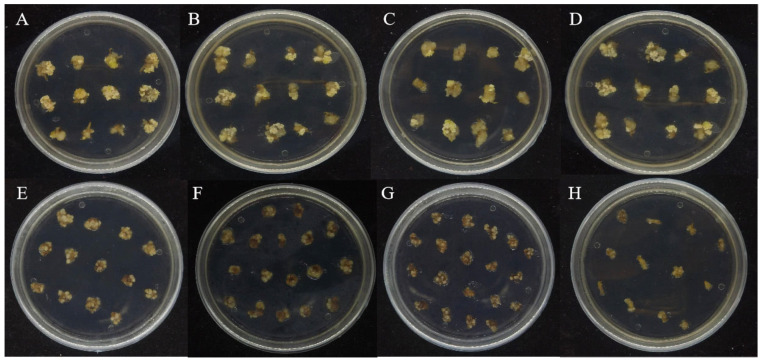
Morphology of calli from foxtail millet varieties after 60 days of induction. (**A**–**D**) High-frequency embryogenic varieties: (**A**) Jingu51; (**B**) Jinpingu1; (**C**) Jinfen17; (**D**) Jingu25. (**E**–**H**) Low-frequency embryogenic varieties: (**E**) Jingu40; (**F**) Jingu29; (**G**) Jingu33; (**H**) Huangjinmiao. Calli from high-frequency varieties produced numerous granular embryogenic calli, whereas those from low-frequency varieties exhibited limited embryogenesis. Scale bar = 1 cm.

Mature embryos from 66 different foxtail millet varieties were inoculated onto CMS to induce callus formation. After 30 days of induction, the resulting primary calli either developed into white or yellow granular structures with the potential to regenerate seedlings (termed embryogenic calli) or failed to do so. Materials exhibiting a high embryogenic frequency typically yielded abundant embryogenic calli, whereas low-frequency materials produced few or no granular calli and often displayed characteristics like browning or water-soaking (Figure 2).

**Figure 3:**
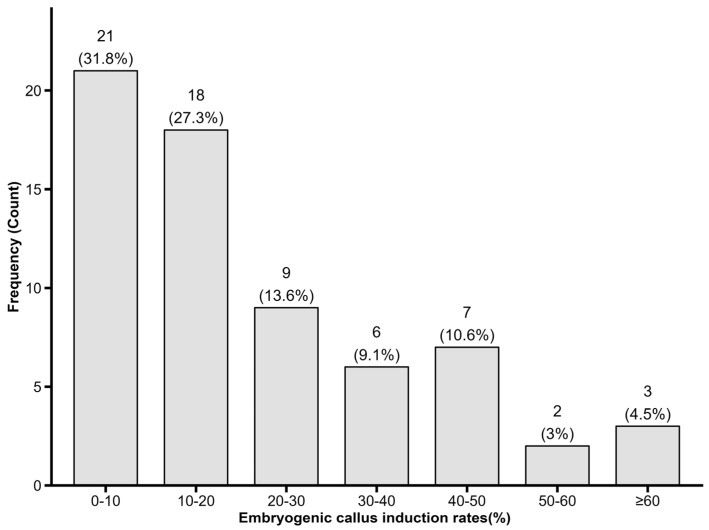
Distribution of embryogenic callus induction rates among 66 foxtail millet varieties. The induction rates represent the mean of three biological replicates, and the varieties are grouped into 10% intervals.

The embryogenic callus induction frequency among the 66 varieties showed a broad range, varying from 0% to 70.22%. Specifically, the distribution of varieties across different frequency ranges was as follows: 21 varieties fell into the 0~10% range; 18 varieties were in the 10~20% range; nine varieties were in the 20~30% range; six varieties were in the 30~40% range; seven varieties were in the 40~50% range; two varieties were in the 50~60% range; and three varieties exceeded 60% (Figure 3, Table S1). Overall, 59.1% of the tested varieties exhibited an embryogenic callus induction frequency below 20%. In contrast, only five varieties—namely Jinpingu1, Jinfen17, Jingu51, Jingu25, and Shanxi Honggu—achieved frequencies above 50%, with specific induction frequencies of 70.22%, 66.07%, 62.77%, 57.81%, and 55.39%, respectively.

**Figure 4:**
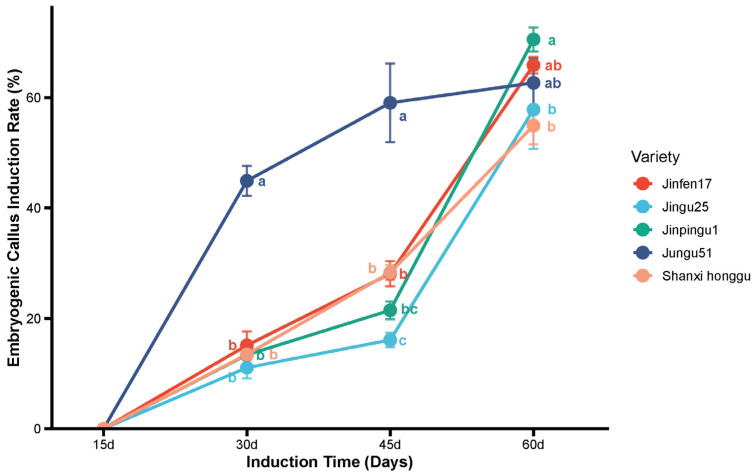
Embryogenic callus induction rates of five high-frequency foxtail millet varieties over 60 days. Embryogenesis was monitored at 0~60 days for Jingu51, Jinfen17, Jinpingu1, Jingu25, and Shanxi Honggu. Jingu51 showed the fastest embryogenic response, reaching ~43% at 30 days and ~60% at 45 days, completing embryogenesis earlier than the other varieties. The data represent the mean ± standard deviation(SD), n = 3 biological replicates. Different letters indicate statistically significant differences according to Tukey HSD following one-way analysis of variance (ANOVA) (*p* < 0.05).

Among these high-frequency varieties, the time course of the embryogenic callus induction rate over 60 days revealed that Jingu51 exhibited the fastest embryogenic response. Although the final embryogenic callus induction rates at 60 days were comparable for all five varieties (range 60~70%), Jingu51 demonstrated a superior initial response: it achieved 43% induction by 30 days and approximately 60% by 45 days. This rapid progression meant that Jingu51 completed the majority of its embryogenesis approximately 15 days earlier than the other varieties (Figure 4). Consequently, due to its exceptionally rapid embryogenic response, Jingu51 was selected as the primary recipient material for all subsequent optimization and genetic transformation experiments.

### 2.2. Effects of Medium Components on Embryogenic Callus Induction

**Figure 5:**
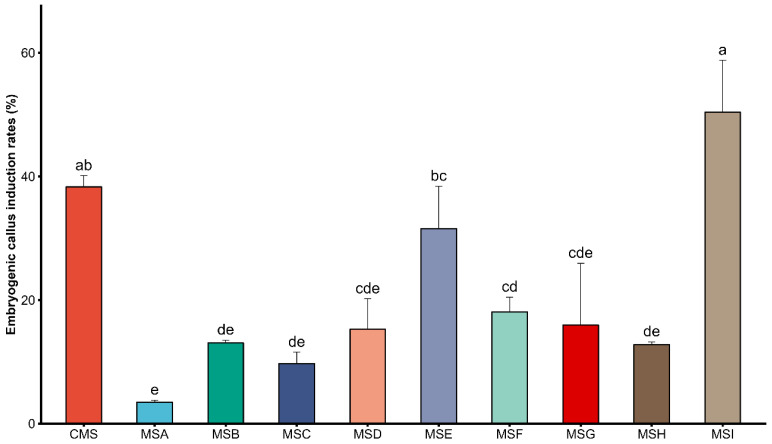
Effects of different medium additives on embryogenic callus induction in foxtail millet. Calli were induced on MS-based media supplemented with various additives. MSA: the basal medium; MSB: MSA plus casein hydrolysate; MSC: MSA plus myo-inositol; MSD: MSA plus ER vitamins; MSE: MSA plus CuSO_4_·5H_2_O; MSF: MSA + plus AgNO_3_; MSG: MSA plus citric acid; MSH: MSA plus AgNO_3_ plus citric acid; MSI: MSA plus casein hydrolysate, myo-inositol, ER vitamins and CuSO_4_·5H_2_O; CMS: standard callus induction medium. The data represent the mean ± SD of three independent replicates. Different letters indicate statistically significant differences according to Tukey HSD following one-way ANOVA (*p* < 0.05).

We reviewed numerous studies on foxtail millet, green foxtail (*Setaria viridis*), maize (*Zea mays*), and rice transformation, and the supplementary components have an important effection to embryogenic callus induction. Adding casein hydrolysate optimized the protocol by enhancing callus regeneration capacity, consistently resulting in multiple shoot formation [9]. Myo-inositol is routinely supplemented in standard plant tissue culture media due to its established role in enhancing plant regeneration [11]. Eriksson’s (ER) vitamin mixture was selected based on its reported benefits in grass species tissue culture [12]. CuSO_4_ was included based on its well-documented stimulatory effects on embryogenesis in wheat (*Triticum aestivum* L.), rice, and triticale (X *Triticosecale* Wittmack) [13,14]. AgNO_3_ was tested as an ethylene inhibitor reported to reduce albinism and increase embryo induction [15]. Citric acid reduces explant browning and enhances shoot growth [16]. In this study, we systematically evaluated the individual contribution of each medium component to embryogenic callus induction and clarified the functional role of these supplements under defined experimental conditions. The supplementation of casein hydrolysate, myo-inositol, ER vitamins, CuSO_4_, AgNO_3_, and citric acid enhanced embryogenic callus induction frequency to varying degrees, with CuSO_4_ exerting the most pronounced effect. The optimized medium MSI (containing 300 mg·L^−1^ casein hydrolysate + 100 mg·L^−1^ myo-inositol + 0.6 mg·L^−1^ CuSO_4_·5H_2_O + 1 mL·L^−1^ ER vitamins) yielded the highest induction rate (Figure 5). Notably, the inclusion of citric acid and AgNO_3_ in the medium led to a decreased induction efficiency.

**Figure 6:**
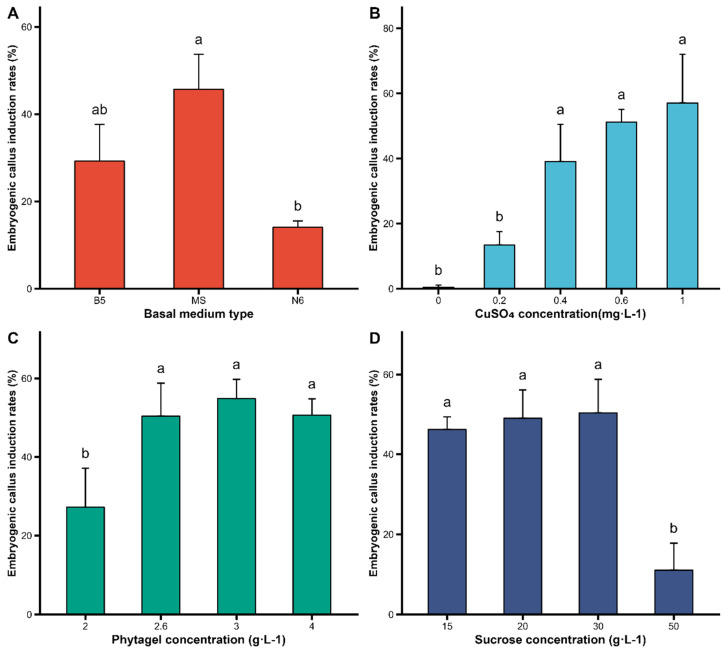
Effects of the main medium components on embryogenic callus induction in foxtail millet. (**A**) Basal medium type (MS, N6, and B5); (**B**) CuSO_4_ concentration (0–1.0 mg·L^−1^); (**C**) phytagel concentration (2.0, 2.6, 3.0, and 4.0 g·L^−1^); (**D**) sucrose concentration (15, 20, 30, and 50 g·L^−1^). The data represent the mean ± SD of three independent replicates. Different letters indicate statistically significant differences according to Tukey HSD following one-way ANOVA (*p* < 0.05).

A comparison of the basal media revealed that the Murashige & Skoog (MS) medium significantly outperformed the CHU (N6) and the Gamborg (B5) medium in promoting callus induction and was thus selected for all subsequent experiments (Figure 6A). The MS medium contains high concentrations of inorganic salts, especially nitrogen sources (nitrate and ammonium), providing essential mineral nutrition that supports robust cell proliferation and stable growth. In contrast, N6 medium provides limited ammonium, and B5 medium contains lower total nitrogen. The balanced macro- and micronutrient composition of MS medium appears to better meet the elevated metabolic demands of embryogenic callus initiation in foxtail millet. Further optimization of individual components revealed that 30 g·L^−1^ sucrose yielded the highest embryogenic induction frequency. Given the critical role of copper (Cu) in this process, supplementation with 1 mg·L^−1^ CuSO_4_ proved optimal. Insufficient copper levels significantly inhibited callus induction (Figure 6B). In comparison to lower concentrations, 3 g·L^−1^ phytagel significantly enhanced embryogenesis (Figure 6C). Sucrose concentrations exceeding 30 g·L^−1^ were significantly detrimental to embryogenic callus induction (Figure 6D).

**Figure 7:**
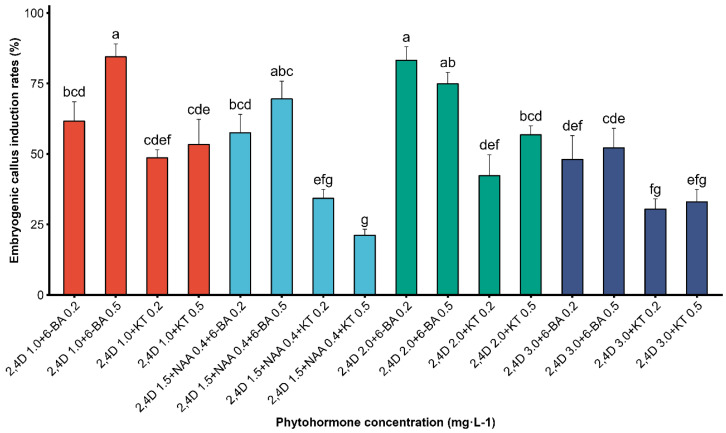
Effects of different phytohormone combinations on embryogenic callus induction in foxtail millet. Calli of Jingu51 were cultured on MS-based media with various combinations of 2,4-D and cytokinin (6-BA or KT). The highest embryogenic callus induction rates were observed with 1 mg·L^−1^ 2,4-D + 0.5 mg·L^−1^ 6-BA and 2 mg·L^−1^ 2,4-D + 0.2 mg·L^−1^ 6-BA, reaching 84.5% and 83.2%, respectively. The data represent the mean ± SD of three independent replicates. Different letters indicate statistically significant differences according to Tukey HSD following one-way ANOVA (*p* < 0.05).

Phytohormone combinations and concentrations significantly influenced the efficiency of embryogenic callus induction. Compared with Kinetin (KT), 6-Benzylamino purine (6-BA) promoted embryogenic callus induction more effectively. The combination of 2,4-Dichlorophenoxyacetic acid (2,4-D) with 6-BA consistently outperformed 2,4-D with KT across all tested concentrations. Under otherwise identical medium conditions, the highest embryogenic callus induction rates for Jingu51 were observed with 1 mg·L^−1^ 2,4-D + 0.5 mg·L^−1^ 6-BA (84.5%) and 2 mg·L^−1^ 2,4-D + 0.2 mg·L^−1^ 6-BA (83.2%), which were not statistically different from each other (*p* > 0.05) (Figure 7).

Based on these results, the optimized callus induction medium for Jingu51 was established as follows: 4.74 g·L^−1^ MS salts with vitamins, 30 g·L^−1^ sucrose, 300 mg·L^−1^ casein hydrolysate, 100 mg·L^−1^ myo-inositol, 1.0 mg·L^−1^ 2,4-D, 0.5 mg·L^−1^ 6-BA, 1 mg·L^−1^ CuSO_4_, 1 mL·L^−1^ ER vitamin solution, 3 g·L^−1^ phytagel, pH 5.8 .

### 2.3. Establishment of Agrobacterium-Mediated Genetic Transformation System of Foxtail Millet

**Figure 8:**
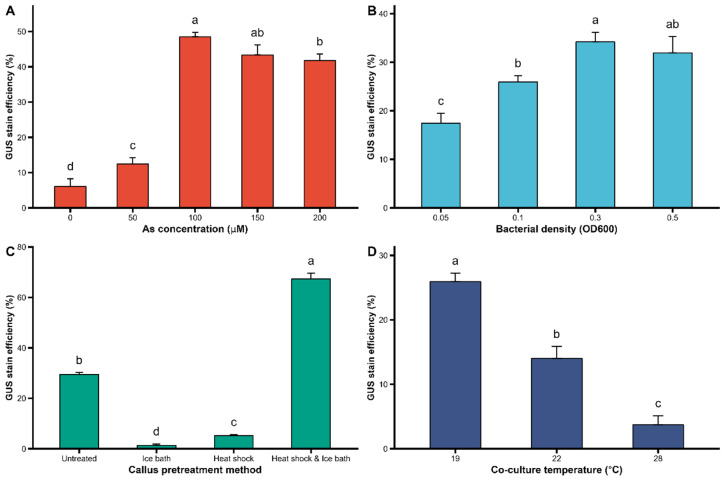
Effects of different infection conditions on *Agrobacterium*-mediated transient transformation efficiency in foxtail millet. (**A**) Acetosyringone concentration; (**B**) *Agrobacterium* suspension density (OD_600_); (**C**) callus pretreatment method; (**D**) coculture temperature. GUS staining efficiency was calculated as the ratio of the blue-stained area to the total surface area of all callus tissues. The data represent the mean ± SD of three independent replicates. Different letters indicate statistically significant differences according to Tukey HSD following one-way ANOVA (*p* < 0.05).

Acetosyringone (AS), a phenolic compound, is crucial for enhancing vir gene expression and improves the efficiency of *Agrobacterium*-mediated transformation. However, its optimal concentration varies significantly across different plant species and even among varieties of the same species. This is particularly relevant for monocotyledonous crops, which fail to produce sufficient endogenous signal inducers, such as acetosyringone, in response to *Agrobacterium* infection. For Jingu51, transformation efficiency increased significantly with acetosyringone concentration, reaching a plateau at 100 μM. Concentrations above 100 μM did not further enhance transformation efficiency (Figure 8A), and thus, 100 μM was selected as the optimal concentration for subsequent experiments.

Bacterial cell density also affected transformation efficiency. Four OD_600_ values (0.05, 0.1, 0.3, and 0.5) were tested, with OD_600_ = 0.3 yielding the highest transformation efficiency. Lower bacterial densities (OD_600_ = 0.05 and 0.1) resulted in insufficient infection, while higher density (OD_600_ = 0.5) led to bacterial overgrowth during cocultivation, which negatively impacted callus recovery and regeneration (Figure 8B).

Callus pretreatment prior to *Agrobacterium* infection significantly influenced transformation efficiency. Four pretreatment methods were compared: no treatment (control); ice bath for 20 min; heat shock at 45 °C for 5 min; and combined heat shock, followed by ice bath. The combined treatment (heat shock at 45 °C for 5 min, immediately followed by ice bath for 20 min) resulted in the highest transformation efficiency, significantly outperforming the other treatments (*p* < 0.05) (Figure 8C). 3,3′-diaminobenzidine (DAB) stain results indicate that heat shock, followed by ice bath, could reduce the reactive oxygen species level obviously (Figure S4).

Cocultivation temperature was found to be a critical parameter for successful transformation. Three temperatures (19, 22, and 28 °C) were evaluated. Notably, the lower temperature of 19 °C resulted in significantly higher transformation efficiency compared to 22 °C and 28 °C (*p* < 0.05) (Figure 8D). The reduced temperature likely inhibits *Agrobacterium* overgrowth while maintaining sufficient vir gene activity, thereby creating a more favorable environment for T-DNA transfer without causing excessive bacterial contamination.

Based on these optimization experiments, the established transformation system for Jingu51 incorporated the following optimal parameters: 100 μM acetosyringone; combined heat shock (45 °C for 5 min), followed by ice bath (20 min) pretreatment; bacterial density of OD_600_ = 0.3; and cocultivation at 19 °C for 2 days in darkness.

**Figure 9:**
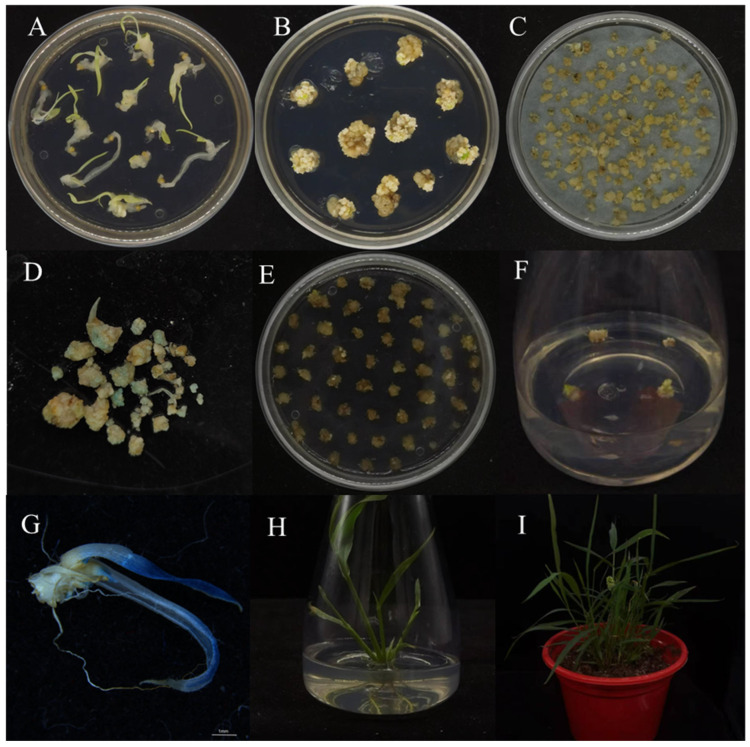
Establishment of the Agrobacterium-mediated genetic transformation system in foxtail millet. (**A**) Primary calli on MSI media after 15 days; (**B**) embryogenic calli of Jingu51; (**C**) calli on coculture media after Agrobacterium infection; (**D**) GUS staining after coculture; (**E**) resistant calli during selection; (**F**) resistant calli on shoot regeneration media; (**G**) GUS staining of transgenic plantlet; (**H**) plantlets on rooting media; (**I**) seedlings transplanted after rooting.

Using embryogenic calli derived from mature seed embryos of Jingu51, a reliable *Agrobacterium*-mediated transformation system was successfully established. The complete transformation procedure was as follows: embryogenic calli were subjected to heat shock at 45 °C for 5 min, immediately transferred to ice for 20 min, and then immersed in *Agrobacterium* suspension (OD_600_ = 0.3, containing 100 μM acetosyringone) for 20 min with gentle agitation. After blotting on sterile filter paper to remove excess bacterial suspension, the infected calli were transferred onto cocultivation medium and cocultured at 19 °C in darkness for 2 days. Following cocultivation, calli were washed with sterile water, blotted dry, and transferred to recovery medium supplemented with 300 mg·L^−1^ timentin and 200 mg·L^−1^ cefotaxime sodium for 7 days in darkness. Recovered calli were then subjected to kanamycin selection medium (50 mg·L^−1^ kanamycin) for approximately 30 days, with subculture every 15~20 days. Kanamycin-resistant calli exhibiting vigorous growth and maintaining embryogenic characteristics were transferred to shoot regeneration medium for approximately 30 days to induce adventitious shoot formation. Shoots exceeding 2 cm in length were excised and transferred to rooting induction medium for approximately 30 days until well-developed root systems formed. Regenerated plantlets with established root systems were carefully transplanted into pots containing sterilized soil and acclimated in a growth chamber under controlled conditions. Transgenic plants grew to maturity and produced viable seeds after 3~4 months of greenhouse cultivation (Figure 9).

### 2.4. Detection of Transgenic Plants

To evaluate the efficiency of the established system, the versatility and robustness of the established protocol were validated by the successful introduction of several additional expression vectors into foxtail millet. The transformation efficiencies for these various constructs ranged from 7.0% to 11.0%, with a mean efficiency of 9.25%; the data was from a single biological replicate (Table 1). These results demonstrate that the optimized system is reproducible for a wide range of genetic engineering applications.

**Figure 10:**
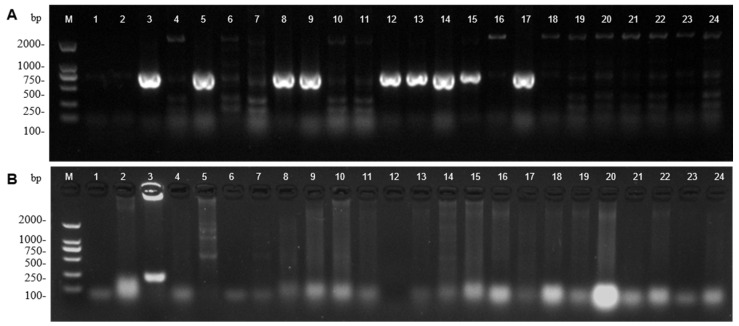
PCR detection of T_0_ generation transgenic foxtail millet plants. (**A**) Target gene *NPTII* detection; (**B**) *virD1* gene detection. M: DNA marker (2000 bp); Lane 1: blank control (ddH_2_O); Lane 2: negative control (wild type); Lane 3: positive control (pBI121/Agrobacterium LBA4404); Lanes 4–24: T_0_ transgenic plants. The expected PCR product size was 798 bp.

PCR analysis confirmed the presence of the transgene in plants (Figure 10A, Table 1, and Figure S1). To confirm the absence of residual *Agrobacterium*, PCR detection of the *virD1* gene was performed simultaneously; no *virD1* signal was detected in any of the surviving plants (Figure 10B and Figure S2). No albino regenerants accounted were observed during the regeneration stage. Three out of 95 plants, including PCR-negative T0 lines, failed to set seeds.

**Figure 11:**
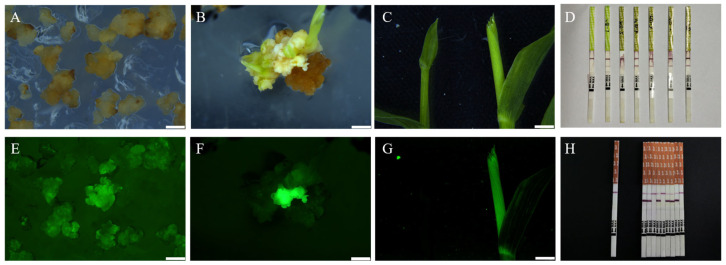
Genetic transformation system application in foxtail millet. (**A**) Calli on coculture medium after Agrobacterium infection (bright field); (**B**) callus on selection medium 30 days after infection (bright field); (**C**) the wild type plant (**left**) and positive plant (**right**) under bright field; (**D**) CP4-EPSPS test strip detection—the left one was wild type, and the six ones on the right were the transgene plants; (**E**) GFP expression in calli on coculture medium after Agrobacterium infection (dark field); (**F**) GFP expression in calli on selection medium 30 days after infection (dark field); (**G**) the wild type plant (**left**) and positive plant (**right**) under dark field; (**H**) *bar* test strip detection—the left one was wild type, and the nine ones on the right were the transgene plants. Imaging parameters (excitation: 470/40 nm; emission: 525/50 nm; exposure time: 500 ms). Scale bar = 2 mm in (**A**–**C**,**E**–**G**).

GFP fluorescence expression in calli and plants was observed under a stereomicroscope, and test strip detection showed the expression of bar or CP4-EPSPS (Figure 11). GFP fluorescence was monitored to identify positive transformation events. During the coculture stage, GFP signals were observed across a large proportion of the callus surface. In the selection stage, bright GFP signals were clearly detected in the proliferating calli. Following regeneration, stable GFP expression was observed in the leaf tissues of T0 plantlets, although the fluorescence intensity appeared relatively weak due to the presence of chlorophyll. The observed fluorescence intensity correlates with the successful integration and expression of the transgene, confirming the efficacy of our optimized transformation protocol.

The T_1_ generation of plants from twelve events were tested using media containing kanamycin or glufosinate-ammonium; a chi-square test (*p* < 0.05) shows 50% of events got single copy after transformation (Table 2).

## 3. Discussion

Foxtail millet is an important minor cereal crop, and significant progress has been made in its breeding and genetic transformation technologies in recent years [9,10]. However, foxtail millet exhibits strong genotype dependence during in vitro culture, and only a limited number of varieties have embryogenic capacity, similar to maize, wheat and sorghum (*Sorghum bicolor*) [17,18]. The large variation observed among the 66 cultivars suggests that embryogenic competence is likely under strong genetic control. Among these cultivars, Jingu51 stands out due to its superior agronomic traits and high regeneration potential, making it an ideal chassis for transgenic breeding and gene-editing applications. Unlike previous studies that utilized callus induction or shoot emergence rates as the primary evaluation criterion [9,10,19], we observed that nearly all embryogenic calli were capable of shoot regeneration on 6-BA-supplemented media. Consequently, we propose that the embryogenic callus induction rate is a more reliable and efficient indicator for identifying high-regeneration germplasm in foxtail millet.

Our results also highlight the critical role of medium additives in modulating embryogenesis. The promotive effect of high CuSO_4_ concentrations on embryogenic callus induction is consistent with observations in *Eleusine coracana*, wheat, and rice [13,14,20]. Copper is a vital micronutrient that serves as a cofactor for numerous important enzymes involved in biological processes, including electron transport, Cu/Zn-SOD activity, and polyphenol oxidase function [21]. The strong response to copper supplementation suggests that micronutrient optimization may be as important as hormonal regulation in determining embryogenic competence [22]. While the underlying mechanisms remain to be fully elucidated, copper and cytokinin potentially improve embryogenesis by activating stress-response pathways and modulating antioxidant enzyme activities (e.g., glutathione S-transferase), which are essential for maintaining redox homeostasis during in vitro morphogenesis [23,24]. However, a high concentration of copper suppressed the formation of embryogenic calli and decreased the regeneration of green plants, suggesting a narrow optimal range for copper supplementation during in vitro culture [25]. Due to important effection in regeneration of plants, further biochemical and molecular analyses are required to confirm these specific mechanisms.

The balance between auxin and cytokinin concentrations in the culture medium plays a decisive role in determining callus morphology and the transition from callus proliferation to somatic embryogenesis. In *Arabidopsis*, exogenous auxin supplementation is typically required to induce embryogenic callus formation, followed by its removal to initiate somatic embryogenesis [26]. In *Citrus*, the requirement for an auxin-depleted environment during initiation appears to precede a later, necessary surge in endogenous IAA, which likely orchestrates subsequent somatic embryo development [27].

The identification of embryogenic calli in this study was primarily based on morphological characteristics and regeneration capacity, and future histological analyses may further validate embryogenic status. Here, we utilized a one-factor-at-a-time approach to optimize the medium composition. While this methodology effectively streamlines the screening process, it overlooks the complex interactions between variables, which remains an important consideration for future experimental refinement.

Plant genetic transformation technologies include two common methods: *Agrobacterium*-mediated transformation and gene gun (biolistic) [28]. Biolistic transformation, while genotype-independent, is associated with high transgene copy numbers, frequent rearrangements, and gene silencing, which complicate downstream functional analysis and breeding applications [28,29,30]. In contrast, *Agrobacterium*-mediated transformation preferentially integrates low copy numbers of T-DNA with defined borders, produces stable single-copy insertion events at higher frequency, and is more amenable to routine laboratory use [31,32]. These advantages make it the most suitable method for establishing a broadly applicable and reproducible transformation platform in foxtail millet, particularly for functional genomics applications requiring predictable transgene expression.

In this study, we established a reproducible transformation system in Jingu51. We identified 19 °C as the optimal coculture temperature, which was consistent with previous findings that elevated temperatures inhibit the assembly of the *Agrobacterium* T4SS (Type IV Secretion System) secretion channel whereas lower temperatures can suppress explant browning and thereby enhance the success rate of T-DNA integration [33,34,35].

Immediate heat exposure prior to *Agrobacterium* infection was effective in the transformation of rice, maize, and sorghum [36,37,38]. Heat shock of plant cells prevents programmed cell death, allowing survival of a greater number of transformed cells following *Agrobacterium* infection [38]. Heat Shock Protein 90.1, which was induced by heat shock, was thought to play an important role in *Agrobacterium*-mediated plant transformation by stabilizing VirE2 interacting protein 1 [39]. Cold pretreatment could reduce browning and was reported in sorghum transformation of immature embryos [40]. Programmed cell death (PCD) may be a factor in transformation efficiency: over-expression of the animal antiapoptosis genes *Bcl-xL* in plant cells inhibited cell death and led to very high transformation efficiency [41]. In this study, we found that a combined physical pretreatment of heat shock and subsequent cold treatment on ice significantly enhanced the *Agrobacterium*-mediated transformation efficiency of foxtail millet (Jingu51). This treatment may integrate the beneficial effects of both heat shock and cold pretreatment, reducing plant cell apoptosis and browning by reducing reactive oxygen species levels.

Foxtail millet has historically been recalcitrant to genetic transformation, a challenge also prevalent in other major crops, such as maize, sorghum, wheat, and rice [42,43]. The transformation efficiency of this study is lower than the highest rates reported by Santos (19.2%) and Sood (26.6%) [9,10]. The present system was validated across four distinct binary vectors, with efficiencies ranging from 7.0% to 11.0%, demonstrating consistent performance across different transgene configurations. Higher efficiencies in those studies were obtained using hygromycin selection system. Our results extend the selection system to kanamycin and glufosinate-ammonium. Glufosinate-ammonium was widely used in the field as an herbicide, so the transgenic plants could be applied to the field directly. Importantly, with previous research often relying on laboratory accessions with limited agronomic value, this work extends investigations to an elite variety. The direct applicability to elite germplasm—rather than to laboratory-adapted accessions—represents the primary practical contribution of this work, as it enables immediate deployment of gene editing and transgenic approaches in a breeding-relevant genetic background (Table 3).

**Table 3:** Comparative analysis of foxtail millet transformation systems with this study.

Study	Material	Tissue Culture Explants	Infection Explants	Total Duration (Weeks)	Selection Strategy	Efficiency
Ceasar 2017 [8]	Maxima	shoot apex	shoot apex	11	25 mg·L^−1^ hygromycin on MRS	9%
Santos 2020 [9]	Yugu1	mature embryo	embryonic calli	17	30 mg·L^−1^ one week on CIM and 8 mg·L^−1^ hygromycin on MRS	19.2%
Sood 2020 [10]	IC-403579; IC-480117	mature embryo	embryogenic calli	12–13	30 mg·L^−1^, followed by 40 mg·L^−1^ hygromycin	26.6%
Yang 2020 [4]	*xiaomi*	mature embryo	yellowish calli	16–20	100 mg·L^−1^ paromomycin or 50 mg·L^−1^ hygromycin	3.08~38.75%
This study	Jingu51	mature embryo	embryonic calli	20	50 mg·L^−1^ kanamycin or 20 mg·L^−1^ glufosinate-ammonium	9.25%

Jingu51 is an elite variety widely cultivated in northern China. It features robust stems with strong drought tolerance and lodging resistance. The panicles are compact and well-filled, with a plant height of 141.2 cm, a panicle length of 21.1 cm, and a panicle weight of 19.9 g. The panicle exhibits a club-shaped morphology with tightly arranged grains. In terms of disease resistance, Jingu51 also performs exceptionally well: it is resistant to blast (*Pyricularia setariae*), moderately resistant to rust (*Uromyces setariae-italicae*), and highly resistant to downy mildew (*Sclerospora graminicola*). In the 2010 regional trials, the average yield was 5098.9 kg/km^2^. The variety is of outstanding grain quality, with bright yellow millet grains and a rich aroma. The protein content of the millet is 11.55%, and the fat content is 3.81%. This achievement effectively bridges the gap between basic laboratory methodology and practical crop improvement. The establishment of a reliable transformation protocol for a leading cultivar provides immediate utility for functional genomics and serves as a powerful tool for the molecular-design breeding of foxtail millet.

Plant regeneration capacity represents a critical determinant of transformation efficiency. Several key embryogenesis-related genes, including *WUSCHEL* (*WUS*) [44], *Baby Boom* (*BBM*) [45], *Growth-Regulating Factor* (*GRF*) [46], and *GRF-Interacting Factor* (*GIF*) [47], have been reported to substantially enhance plant transformation efficiency either individually or in combination [17,47,48]. However, such molecular strategies have yet to be extensively applied to foxtail millet. In future studies, we will investigate the roles of various regeneration-promoting factors in the genetic transformation system of this crop.

The establishment of a transformation platform in an elite cultivar creates opportunities for the direct deployment of genome editing technologies in breeding programs.

## 4. Materials and Methods

### 4.1. Plant Materials

A total of 66 cultivated foxtail millet varieties were used to screen for genotypes with high embryogenic capacity in this study (Table S1). All varieties are registered cultivars or breeding lines obtained from collaborative breeding programs with Dr. Kai Zhao (College of Agriculture, Shanxi Agricultural University) (34 varieties), Dr. Chen-yuan Yang (The Industrial Crop Institute, Shanxi Agricultural University) (4 varieties), and Dr. Jun Wang (Millet Research Institute, Shanxi Agricultural University) (28 varieties).

### 4.2. Seed Sterilization

Foxtail millet seeds were first washed thoroughly to remove shriveled grains. Surface sterilization was subsequently performed in a laminar flow hood by immersing the seeds in 75% (*v*/*v*) ethanol for 45 s, followed by treatment with 10% (*v*/*v*) sodium hypochlorite solution for 15–20 min. The seeds were then rinsed 3–5 times with sterile distilled water, 1 min per rinse. Finally, the sterilized seeds were placed on sterile filter paper to remove excess moisture.

### 4.3. Screening of High-Frequency Embryogenic Varieties of Foxtail Millet

Sterilized seeds from all 66 varieties were placed onto callus induction medium (CMS) as described by Santos [9]. For each variety, 100 seeds were randomly selected as a biological replicate, with 10–15 seeds per dish; each variety consisted of three biological replicates. Cultures were maintained in a growth chamber at 23 °C in darkness. Calli were subcultured onto fresh CMS every 20 days. After two rounds of subculture, light yellow, compact embryogenic calli typically emerged at the margins or apical regions of the primary callus. Viability was assessed based on the maintenance of a compact, nodular, and creamy-yellow appearance, as these morphological characteristics are strongly correlated with high embryogenic potential in *Setaria italica*.

Seed germination rate was recorded 7 days after inoculation. Subsequently, the number of embryogenic calli was scored at each subculture interval until day 60. The following parameters were calculated:

$$Germination\;rate(\%)=\frac{Number\;of\;germinated\;seeds}{Total\;number\;of\;inoculated\;seeds}\times 100$$

$$Embryogenic\;calli\;induction\;rates\left(\%\right)=\frac{Number\;of\;embryogenic\;calli}{Number\;of\;germinated\;seeds}\times 100$$

Embryogenic calli were transferred to shoot regeneration medium (MRS). Adventitious shoots exceeding 2 cm in height were subsequently subcultured onto rooting induction medium (RIM) to facilitate root development (Table 4). Once well-developed root systems were established, the plantlets were removed from the culture vessels, gently rinsed to remove residual media, and transplanted into pots containing a soil mixture of vermiculite and peat moss (3:1, *v*/*v*). Plants were maintained in a growth chamber (26 °C; 14 h light/10 h dark photoperiod) and irrigated regularly.

### 4.4. Optimization of the Callus Induction Medium

In this study, the key components of the CMS medium were systematically optimized to determine the most effective formulation for embryogenic callus induction. First, three basal media—MS salts (with vitamins) [49], N6 salts (with vitamins) [50], and B5 salts (with vitamins) [51]—were compared to identify the optimal basic medium.

Based on a basic medium (designated MSA) formulation containing MS salts with vitamins, phytohormones, and phytagel, we systematically evaluated the impact of individual supplements, including 300 mg·L^−1^ casein hydrolysate, 100 mg·L^−1^ myo-inositol, 1 mL·L^−1^ of ER vitamins (0.5 g·L^−1^ VB_1_, 0.5 g·L^−1^ VB6, 0.5 g·L^−1^ nicotinic acid, and 2 g·L^−1^ glycine) [12], 0.6 mg·L^−1^ CuSO_4_, 2 mg·L^−1^ AgNO_3_, and 1 mg·L^−1^ citric acid (Table 5).

Subsequently, concentration-response experiments were conducted to determine the optimal levels of sucrose (15, 20, 30, and 50 g·L^−1^), copper sulfate (0, 0.2, 0.4, 0.6, and 1.0 mg·L^−1^), and phytagel (2.0, 2.6, 3.0, and 4.0 g·L^−1^).

Finally, the optimal concentration and combination of phytohormones were investi-gated. Various ratios of 2,4-D (1.0~3.0 mg·L^−1^), KT (0.2~0.5 mg·L^−1^), and 6-BA (0.2~0.5 mg·L^−1^) were tested to evaluate their synergistic effects on callus induction.

Each treatment consisted of three biological replicates, with 30 inoculated seeds randomly selected per replicate. The frequency of embryogenic callus formation within 60 days was used as the evaluation parameter. Data are presented as mean ± SD, and differences were considered statistically significant at *p* < 0.05. Data were analyzed using one-way ANOVA, followed by Tukey HSD for multiple comparisons. The homogeneity of variance was verified using Levene’s test. All statistical analyses were conducted in R(version 4.3.1, R Foundation for Statistical Computing, Vienna, Austria), with statistical significance set at *p* < 0.05.

### 4.5. Establishment of Agrobacterium-Mediated Transformation System of Foxtail Millet

*Agrobacterium tumefaciens* strain LBA4404 harboring the binary vector *pBI121 (gus* as marker gene and *nptII* as the selection gene) was streaked onto LB agar plates supplemented with 50 mg·L^−1^ kanamycin and 25 mg·L^−1^ rifampicin and cultured at 28 °C for 48 h. Single colonies were inoculated into liquid LB medium and grown at 28 °C with shaking (180 rpm) until the culture reached an OD_600_ of 0.6. Bacterial cells were harvested by centrifugation at 4500 rpm for 10 min at room temperature. The supernatant was discarded, and the bacterial pellet was resuspended in infection medium (Table 6) and adjusted to an OD_600_ of 0.3. Each treatment consisted of three biological replicates, with 100 calli chosen randomly per replicate.

The fresh compact embryogenic calli were pretreated by heat shock at 45 °C for 5 min, immediately transferred to ice for 20 min, and then immersed in the *Agrobacterium* suspension for 20 min with gentle agitation. Infected calli were blotted dry on sterile filter paper, transferred onto cocultivation medium overlaid with sterile filter paper, and cocultured in darkness at the specified temperature for 2 days. Following cocultivation, the calli were washed 5–6 times with sterile water containing 500 mg·L^−1^ cefotaxime to remove residual bacteria, blotted dry, and transferred to recovery medium for 7 days in darkness (Table 6).

Following recovery, calli were transferred to selection medium containing 50 mg·L^−1^ kanamycin and 500 mg·L^−1^ cefotaxime and subcultured every 15–20 days for a total selection period of 30 days. Kanamycin-resistant calli exhibiting continued growth and maintaining embryogenic characteristics were transferred to MRS for adventitious shoot induction. Shoots exceeding 2 cm in length were excised and transferred to RIM for root development (Table 6).

### 4.6. GUS Stain

To optimize transformation parameters, the effects of acetosyringone concentration (0, 50, 100, 150, and 200 μM), *Agrobacterium* bacterial density (OD_600_ = 0.05, 0.1, 0.3, and 0.5), callus pretreatment methods (no treatment; ice bath for 20 min; heat shock at 45 °C for 5 min; or combined heat shock at 45 °C for 5 min, followed by ice bath for 20 min), and cocultivation temperature (19, 22, and 28 °C) on transformation efficiency were systematically evaluated using GUS histochemical staining. Each experiment was independently repeated three times, with each replicate consisting of 60 embryogenic calli.

Following cocultivation, calli were subjected to GUS histochemical staining at 37 °C for 24 h using a GUS staining kit (Cat: G3060, Beijing Solarbio Life Sciences, Co., Ltd., China). After staining, tissues were decolorized with 75% (*v*/*v*) ethanol to remove chlorophyll and enhance color contrast. GUS-positive (blue) areas were visualized and photographed under a stereomicroscope. The proportion of stained area relative to total callus area was quantified using ImageJ software (version 1.8.0, NIH, Bethesda, Maryland, USA). Images were acquired under standardized conditions (fixed magnification, white balance, and light intensity). Prior to analysis, all images were converted to 8-bit grayscale, and a consistent threshold was applied based on the color contrast between GUS-stained (blue) and unstained (white/cream) callus tissue. The proportion of GUS-stained area relative to total callus area was calculated using the ‘Analyze Particles’ function.

Transient transformation efficiency was evaluated as the percentage of the GUS-stained area relative to the total callus area, as determined by image analysis. Statistical analyses were performed using one-way ANOVA, followed by Tukey’s HSD post hoc test. Homogeneity of variance was assessed using Levene’s test. All statistical analyses were conducted in R (version 4.3.1, R Foundation for Statistical Computing, Vienna, Austria). Data are presented as mean ± standard deviation (SD), and differences were considered statistically significant at *p* < 0.05.

### 4.7. DNA Extract and PCR

Genomic DNA was extracted from leaf tissue of putative transgenic plants using a CTAB method [53]. PCR amplification of the *nptII* gene was performed to confirm transgene integration. The primers used were: NptII-F (5′-ATGGGGATTGAACAAGATGGATTG-3′) and NptII-R (5′-TCAGAAGAACTCGTCAAGAAGGCG-3′), yielding an expected amplicon of 798 bp. PCR reactions were carried out in a total volume of 25 μL containing 50 ng template DNA, 0.4 μM of each primer, and 12.5 μL 2× Taq PCR Master Mix (Cat: KT201, Tiangen Biotech (Beijing) Co., Ltd., Beijing, China). Amplification conditions were as follows: initial denaturation at 94 °C for 2 min, followed by 32 cycles of denaturation at 94 °C for 15 s, annealing at 60 °C for 20 s, and extension at 72 °C for 1 min, with a final extension at 72 °C for 5 min. PCR products were separated by electrophoresis on 1.5% (*w*/*v*) agarose gels in 1× TAE buffer at 120 V for 30 min, stained with ethidium bromide or a safer alternative, and visualized under UV light. A DNA Marker 2000 (Cat: B500350, Sangon Biotech (Shanghai) Co., Ltd., Shanghai, China) was used as a molecular weight marker.

To exclude Agrobacterium contamination, the presence of the *virD1* gene in putative transgenic plants was examined by PCR. The primers used were: VirD1-F (5′-GGGTTCAAGGTCGTGAGTGC-3′) and VirD1-R (5′-TAGAAGCATGGATACATTGC-3′), yielding an expected amplicon of 213 bp [54].

$$Stable\;transformation\;efficiency\left(\%\right)=\frac{PCR\;positive\;plants}{Infected\;calli}\times 100$$

### 4.8. Vector Information

We also used other vectors to confirm the robustness of this foxtail millet transformation system. The vectors include: pCAMBIA3301 (including *gus* as the marker gene and *bar* as the selection gene), p3341-CP4-EPSPS (including *CP4-EPSPS* and *bar* as the selection gene), and p3300-GFP (including *GFP* as the marker gene and *bar* as the selection gene).

### 4.9. GFP Fluorescence Detection

GFP fluorescence was visualized using a Leica M205 FCA(Leica Microsystems (Schweiz) AG, Heerbrugg, Switzerland) fluorescence stereomicroscope equipped with appropriate filter sets (excitation: 470/40 nm; emission: 525/50 nm). Images were captured with an exposure time of 500 ms and a gain value of 10. GFP expression was monitored in calli at 3 days post-inoculation (dpi) on cocultivation medium, at 30 dpi on selection medium, and in confirmed transgenic plantlets.

### 4.10. Lateral Flow Test

Transgenic plants harboring either the *bar* gene (conferring glufosinate resistance) or the *CP4-EPSPS* gene (conferring glyphosate resistance) were identified using commercially available lateral flow test strips specific for the respective proteins, following the manufacturer’s instructions.

### 4.11. Segregation Ratios in the T_1_ Generation of Plants

The T_1_ generation of seeds were sterilized and then placed on the identification medium (Table 6). Seven days later, the resistant and sensitive plants were identified.

## 5. Conclusions

Among the 66 foxtail millet varieties screened in this study, five were identified as possessing high embryogenic potential, with Jingu51 selected as the primary recipient for system optimization. By synergistically combining CuSO_4_ supplementation with optimized phytohormone ratios (specifically 2,4-D and 6-BA), we significantly enhanced embryogenic callus induction and quality. Furthermore, through the fine-tuning of *Agrobacterium*-mediated infection parameters, a stable and reproducible transformation system was established, achieving a mean efficiency of 9.25% in different vectors; half transgenic event exhibited single copy confirmed by *χ*^2^ goodness-of-fit tests (*p* > 0.05). The successful implementation of this protocol in Jingu51—a locally adapted elite cultivar combining high drought tolerance with strong lodging resistance—represents a practical and reproducible contribution to the genetic transformation toolbox for foxtail millet. Crucially, by establishing a validated transformation system directly in a variety already favored by farmers, this work provides a direct pathway for introducing quality-improvement and stress-tolerance genes into agronomically relevant germplasm, thereby accelerating the biotechnological improvement of foxtail millet in real-world breeding programs.

## Figures and Tables

**Table 1:** Genetic transformation efficiency of foxtail millet using different vectors.

Vector	Resistance Gene	Number of Infected Calli	Number of Surviving Calli	Number of Surviving Seedlings	Number of PCR-Positive Plants	Transformation Efficiency
pBI121	*NptII*	300	107	60	21	7%
p3301	*Bar*	100	21	14	10	10%
p3300-CP4-EPSPS	*Bar*	100	13	9	9	9%
p3300-GFP	*Bar*	100	15	12	11	11%

Note: Transformation efficiency (%) = number of PCR-positive T_0_ plants ÷ total number of infected embryogenic calli × 100. Each value represents a single independent transformation experiment.

**Table 2:** Segregation ratios in the T_1_ generation of plants transformed with pBI121 and p3300-GFP.

Vector	Line	Total	Resistant	Sensitive	*p*-Value	*X^2^* Value for 3:1	Fits 3:1 Ratio
pBI121 ^a^	2	58	48	10	0.172	1.862	Yes
pBI121	5	56	39	17	0.355	0.857	Yes
pBI121	6	58	50	8	0.049	3.885	No
pBI121	9	56	32	24	0.002	9.524	No
p3300-GFP ^b^	1	84	71	13	0.044	4.063	No
p3300-GFP	2	56	50	6	0.014	6.095	No
p3300-GFP	3	56	44	12	0.537	0.381	Yes
p3300-GFP	4	70	67	3	6.271 × 10^−5^	16.019	No
p3300-GFP	5	56	48	8	0.064	3.429	Yes
p3300-GFP	6	59	40	19	0.201	1.633	Yes
p3300-GFP	7	55	41	14	0.938	0.006	Yes
p3300-GFP	8	43	22	21	3.063 × 10^−4^	13.031	No

Note: ^a^ number of resistant seedlings on medium containing 20 mg L^−1^ kanamycin; ^b^ number of resistant seedlings on medium containing 10 mg L^−1^ glufosinate-ammonium.

**Table 4:** Culture media used in the regeneration experiments.

Medium	Medium Composition
Callus induction medium (CMS) [9]	4.74 g·L^−1^ MS salts (including vitamins) + 300 mg·L^−1^ casein hydrolysate + 100 mg·L^−1^ myo-inositol + 0.6 mg·L^−1^ CuSO_4_ + 30 g·L^−1^ sucrose + 2 mg·L^−1^ 2,4-D + 0.5 mg·L^−1^ KT + 2.6 g·L^−1^ phytagel +2 mg·L^−1^ AgNO3 + 1 mg·L^−1^ citric acid, pH 5.8
Optimized CMS	4.74 g·L^−1^ MS salts (including vitamins) + 300 mg·L^−1^ casein hydrolysate + 100 mg·L^−1^ myo-inositol + 1.0 mg·L^−1^ CuSO_4_ + 30 g·L^−1^ sucrose + 1 mL·L^−1^ ER vitamin solution + 1 mg·L^−1^ 2,4-D + 0.5 mg·L^−1^ 6-BA + 3.0 g·L^−1^ phytagel, pH 5.8
Regeneration medium (MRS) [9]	4.74 g·L^−1^ MS salts (including vitamins) + 300 mg·L^−1^ casein hydrolysate + 0.6 mg·L^−1^ CuSO_4_ + 30 g·L^−1^ sucrose + 1 mg·L^−1^ 6-BA+ 2.6 g·L^−1^ phytagel, pH 5.8
Rooting induction medium (RIM) [4]	2.37 g·L^−1^ MS salts (including vitamins) + 30 g·L^−1^ sucrose + 100 mg·L^−1^ myo-inositol + 0.1 mg·L^−1^ IBA + 2.6 g·L^−1^ phytagel, pH 5.8

Note: MS salts (including vitamins) (Cat: M0222, Duchefa Biochemie B.V., Haarlem, The Netherlands), phytagel (Cat: G434, PhytoTechnology Laboratories, Lenexa, KS, USA), casein hydrolysate (Cat: C8221, Beijing Solarbio Life Sciences, Co., Ltd., Beijing, China), myo-inositol (Cat: I8050, Beijing Solarbio Life Sciences, Co., Ltd., Beijing, China), timentin (Cat: CT11182, Coolaber Science and Technology, Beijing, China). ER vitamin solution contains thiamine hydrochloride 0.5 g·L^−1^, pyridoxine hydrochloride 0.5 g·L^−1^, nicotinic acid 0.5 g·L^−1^ and glycine 2 g·L^−1^.

**Table 5:** Composition of media used for optimizing callus induction.

Medium	Medium Composition
MSA	4.74 g·L^−1^ MS salts with vitamins + 30 g·L^−1^ sucrose + 2.0 mg·L^−1^ 2,4-D + 0.5 mg·L^−1^ KT + 2.6 g·L^−1^ phytagel, pH 5.8
MSB	MSA + 300 mg·L^−1^ casein hydrolysate
MSC	MSA + 100 mg·L^−1^ myo-inositol
MSD	MSA + 1 mL·L^−1^ ER vitamins
MSE	MSA + 0.6 mg·L^−1^ CuSO_4_·5H_2_O
MSF	MSA + 2 mg·L^−1^ AgNO_3_
MSG	MSA + 1 mg·L^−1^ citric acid
MSH	MSA + 2 mg·L^−1^ AgNO_3_ + 1 mg·L^−1^ citric acid
MSI	MSA + 300 mg·L^−1^ casein hydrolysate + 100 mg·L^−1^ myo-inositol + 1 mL·L^−1^ ER vitamins + 0.6 mg·L^−1^ CuSO_4_·5H_2_O

**Table 6:** Culture media used in the transformation experiment.

Medium	Medium Composition
Infection medium [52]	2 g·L^−1^ N6 salts, 68.5 g·L^−1^ sucrose, 36 g·L^−1^ glucose, 0.7 g·L^−1^ L-proline, 2 mg·L^−1^ 2,4-D, 0.5 g·L^−1^ MES, pH 5.5 (100 μM acetosyringone)
Coculture medium	4.74 g·L^−1^ MS salts (including vitamins), 30 g·L^−1^ sucrose, 30 g·L^−1^ glucose, 100 mg·L^−1^ L-glutamine, 100 mg·L^−1^ L-asparagine, 100 mg·L^−1^ L-cysteine + 2 mg·L^−1^ 2,4-D + 0.5 g·L^−1^ MES + 2.6 g·L^−1^ phytagel, pH 5.5 (100 μM·acetosyringone)
Recovery medium	4.74 g·L^−1^ MS salts (including vitamins) + 300 mg·L^−1^ casein hydrolysate + 100 mg·L^−1^ myo-inositol + 0.6 mg·L^−1^ CuSO_4_·5H2O + 30 g·L^−1^ sucrose + 1 mg·L^−1^ 2,4-D + 0.5 mg·L^−1^ 6-BA + 3 g·L^−1^ phytagel, pH 5.8(300 mg·L^−1^ timentin *)
Selection medium	4.74 g·L^−1^ MS salts (including vitamins) + 300 mg·L^−1^ casein hydrolysate + 100 mg·L^−1^ myo-inositol + 0.6 mg·L^−1^ CuSO_4_·5H2O + 30 g·L^−1^ sucrose + 1 mg·L^−1^ 2,4-D + 0.5 mg·L^−1^ 6-BA + 3 g·L^−1^ phytagel, pH 5.8 (supplemented with 300 mg·L^−1^ timentin + 50 mg·L^−1^ kanamycin/20 mg·L^−1^ glufosinate-ammonium)
Regeneration medium	4.74 g·L^−1^ MS salts (including vitamins) + 300 mg·L^−1^ casein hydrolysate + 0.6 mg·L^−1^ CuSO_4_·5H2O + 30 g·L^−1^ sucrose + 1 mg·L^−1^ 6-BA + 2.6 g·L^−1^ phytagel, pH 5.8 (supplemented with 300 mg·L^−1^ timentin + 50 mg·L^−1^ kanamycin/10 mg·L^−1^ glufosinate-ammonium)
Rooting induction medium	2.37 g·L^−1^ MS salts (including vitamins) + 30 g·L^−1^ Sucrose + 100 mg·L^−1^ myo-inositol + 0.1 mg·L^−1^ IBA + 2.6 g·L^−1^ phytagel, pH 5.8
Identification medium	2.37 g·L^−1^ MS salts (including vitamins) + 30 g·L^−1^ Sucrose + 100 mg·L^−1^ myo-inositol + 2.6 g·L^−1^ phytagel, pH 5.8 (supplemented with 50 mg·L^−1^ kanamycin/10 mg·L^−1^ glufosinate-ammonium)

Note: * Timentin (Cat: CT11182, Coolaber Science and Technology, Beijing, China).

## Data Availability

The original contributions presented in this study are included in the article/Supplementary Materials. Further inquiries can be directed to the corresponding author.

## Supplementary Materials

The following supporting information can be downloaded at: https://www.mdpi.com/article/10.3390/plants15142159/s1, Figure S1: Complete original gel images for PCR verification of transgenic foxtail millet plants; Figure S2: Complete original gel images for PCR detection for vir gene of transgenic foxtail millet plants; Table S1: Callus induction data for 66 foxtail millet varieties; Figure S3: Effects of different infection conditions on Agrobacterium-mediated transient transformation efficiency in foxtail millet by GUS stain; Figure S4: DAB stain after calli pretreatment.
