# Supplementary material for: Efficient Embryogenic Callus Induction and Agrobacterium-Mediated Transformation of the Elite Foxtail Millet (Setaria italica L.) Variety Jingu51
Source: Plants (Basel). 2026 Jul 13;15(14):2159. doi: 10.3390/plants15142159 (PMC13414878; doi:10.3390/plants15142159)
Supplement: Supplementary file 1 [file plants-15-02159-s001.zip › plants-4386856-supplementary Figures.pdf]

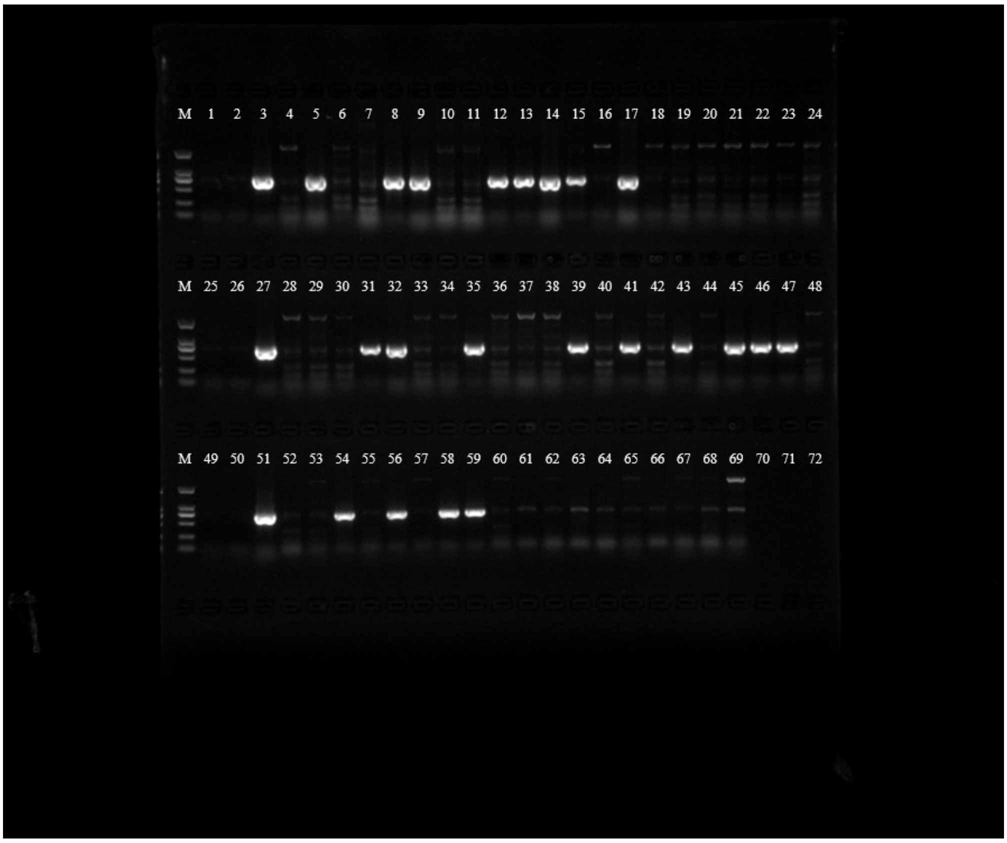

Figure S1 Complete original gel images for PCR verification of transgenic foxtail millet plants. M: DNA marker (2000 bp); Lane 1,25 and 49: blank control (ddH_2O); Lane 2,26 and 50: negative control (wild type); Lane 3, 27 and 51: Positive control (pBI121 Vector); Lane4-24,28-48 and 52-69: T_0 transgenic plants. The expected PCR product size was 798 bp.

PCR conditions:
Template DNA: 50 ng genomic DNA
Primers: nptII-F/R (as described in Methods)
Program: 94°C 2 min; 32 cycles (94°C 15s, 60°C 20s, 72°C 1 min); 72°C 5 min - Expected product: 798 bp
Date: February 8, 2026
Positive Lanes: 5, 8, 9, 12, 13, 14, 15, 17, 31, 32, 35, 39, 41, 43, 45, 46, 47, 54, 56, 58, 59

Summary:
Total plantlets tested: 60
PCR-positive: 21 (35%)
PCR-negative (escapes): 39 (65%)
Transformation efficiency: 21/300 = 7%

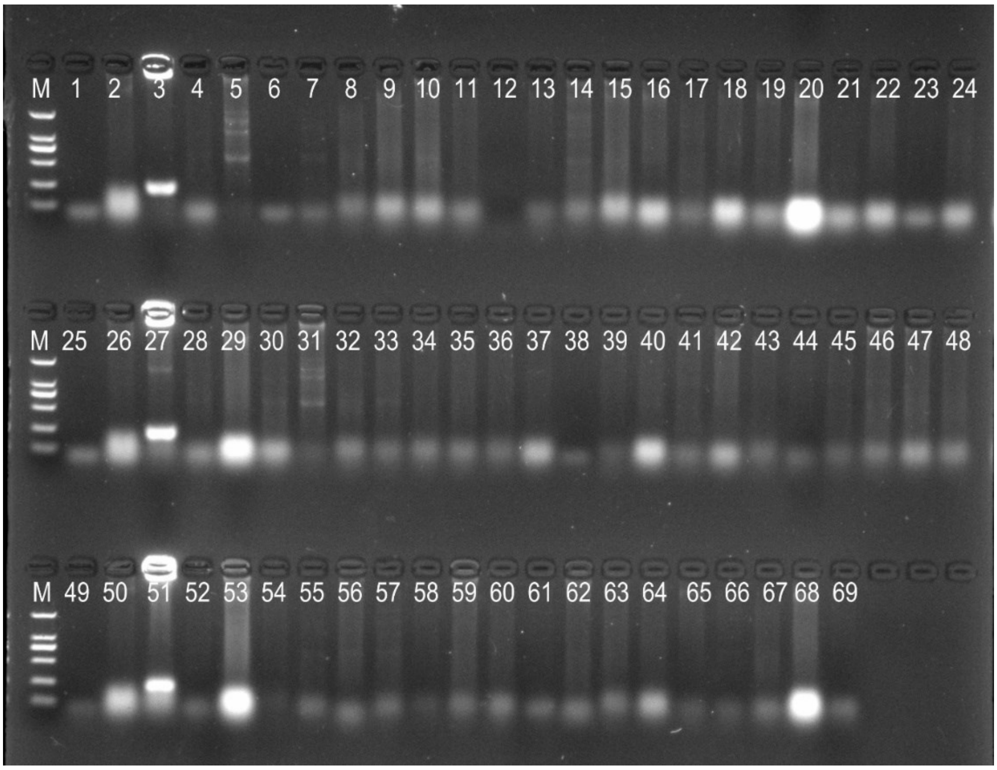

Figure S2 Complete original gel images for PCR detection for vir gene of transgenic foxtail millet plants. M: DNA marker (2000 bp); Lane 1,25 and 49: blank control (ddH2O); Lane 2,26 and 50: negative control (wild type); Lane 3, 27 and 51: Positive control (Agrobacterium tumefaciens strain LBA4404); Lane4-24,28-48 and 52-69: T0 transgenic plants. The expected PCR product size was 213 bp.

PCR conditions:

Template DNA: 50 ng genomic DNA

Primers: virD1-F/R (as described in Methods)

Program: 94°C 2 min; 32 cycles (94°C 15s, 60°C 20s, 72°C 30s); 72°C 5 min - Expected product: 213 bp

Date: May 25, 2026

Positive Lanes: No detected.

Summary:

Total plantlets tested: 60

The size of band of Positive control was as expected.

No plants showed detectable Agrobacterium contamination.

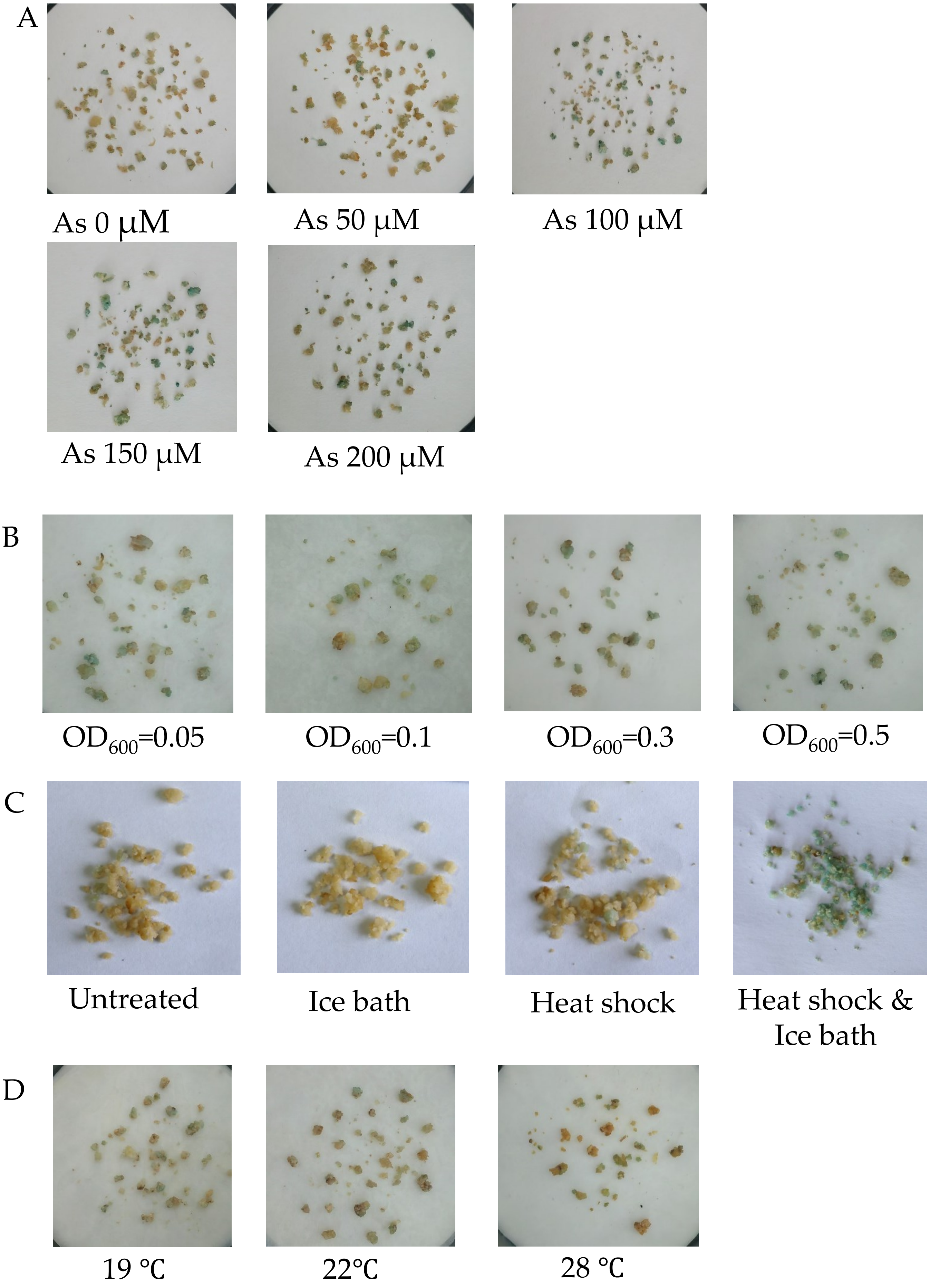

Figure S3. Effects of different infection conditions on *Agrobacterium*-mediated transient transformation efficiency in foxtail millet by GUS stain. (A) Acetosyringone concentration; (B) Agrobacterium suspension density (OD_{600}); (C) callus pretreatment method; (D) coculture temperature.

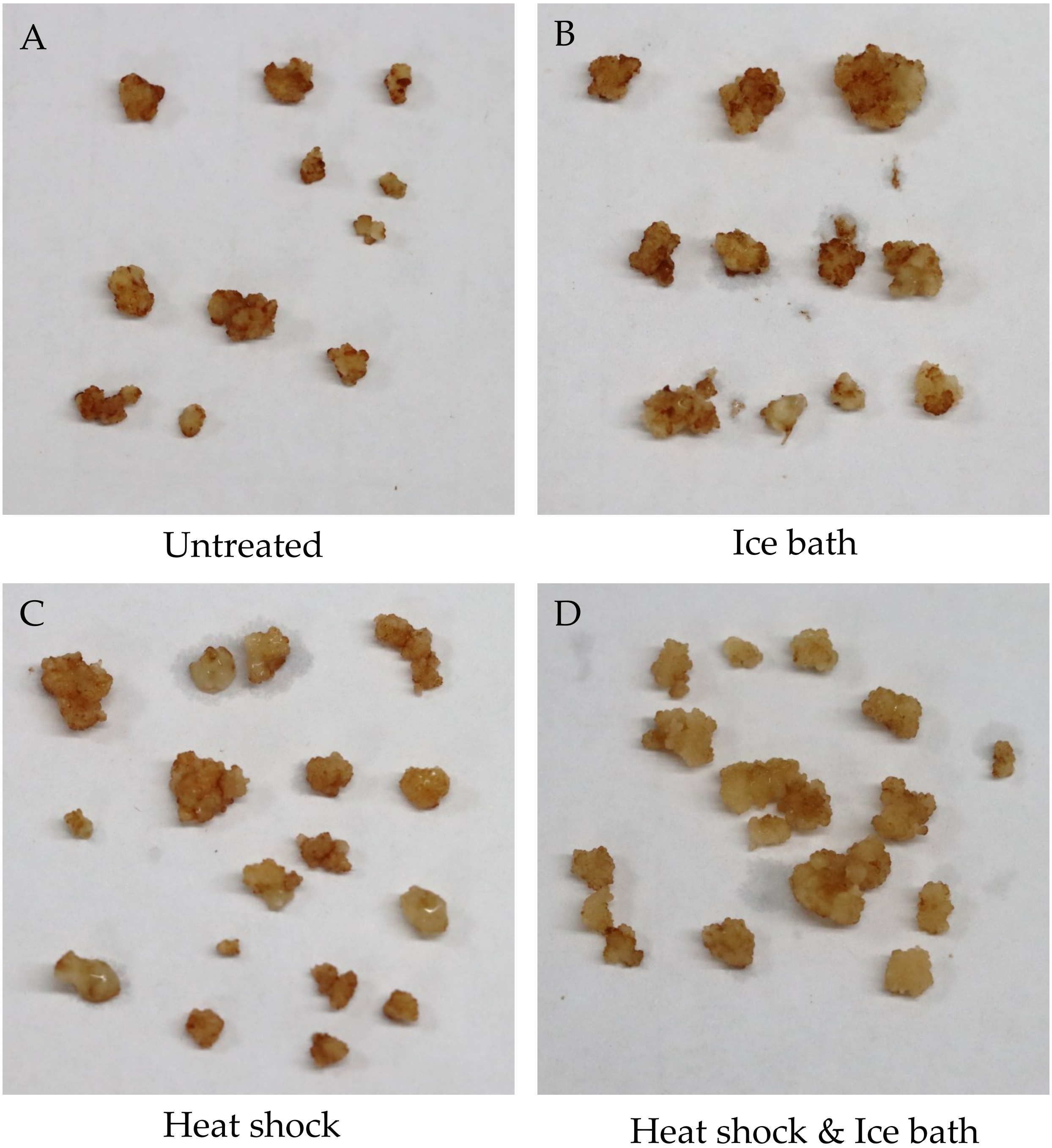

Figure S4. DAB stain after calli pretreatment. (A) Untreated calli; (B) Calli pretreated in ice bath 20 mins; (C) Calli heat shocked in 45 °C for 5 mins; (D) Calli heat shocked in 45 °C for 5 mins and followed by ice bath 20 mins.
