# Supplementary material for: Efficient Embryogenic Callus Induction and Agrobacterium-Mediated Transformation of the Elite Foxtail Millet (Setaria italica L.) Variety Jingu51
Source: Plants (Basel). 2026 Jul 13;15(14):2159. doi: 10.3390/plants15142159 (PMC13414878; doi:10.3390/plants15142159)
Supplement: Supplementary file 1 [file plants-15-02159-s001.zip › Supplementary Table S1.pdf]

Table S1. 66 foxtail millet varieties callus induction data

Name	Source	Germination rate ± SD (%)	Embryogenic calli induction rate ± SD (%)
Jingu51	K. Zhao	83.02 ± 3.36	62.77 ± 3.75
Jingu21	K. Zhao	96.65 ± 4.15	12.61 ± 1.34
Yugu35	K. Zhao	76.75 ± 3.27	11.77 ± 2.95
Taixuangu22	K. Zhao	74.44 ± 1.24	1.75 ± 0.73
Taixuangu24	K. Zhao	86.01 ± 5.19	33.71 ± 3.17
Jingu34	K. Zhao	93.01 ± 1.57	34.65 ± 4.11
Jingu36	K. Zhao	94.29 ± 3.56	16.86 ± 5.17
Jingu60	K. Zhao	83.61 ± 1.08	15.81 ± 7.55
Jingu62	K. Zhao	78.84 ± 1.00	24.57 ± 4.21
Jingu52	K. Zhao	76.64 ± 4.04	17.13 ± 2.74
Jigu39	K. Zhao	71.07 ± 2.56	2.01 ± 0.94
Jigu41	K. Zhao	68.62 ± 2.40	9.08 ± 2.34
Jigu168	K. Zhao	96.74 ± 3.01	43.04 ± 3.59
Yugu18	K. Zhao	85.69 ± 9.09	15.01 ± 1.97
Jiugu23	K. Zhao	85.54 ± 4.22	17.39 ± 3.60
Zhonggu9	K. Zhao	97.78 ± 1.94	27.78 ± 1.36
Changnong35	K. Zhao	98.98 ± 1.03	20.52 ± 3.97
Jigu22	K. Zhao	95.84 ± 5.49	28.56 ± 2.55
Nenxuan15	K. Zhao	95.82 ± 4.44	27.59 ± 0.34
Zhonggu2	K. Zhao	94.76 ± 6.17	26.02 ± 1.76
ChiyouJinmiao1	K. Zhao	97.31 ± 3.05	27.77 ± 1.28
Zhangzagu16	K. Zhao	97.84 ± 1.90	1.92 ± 0.90
Longgu25	K. Zhao	92.67 ± 3.93	37.56 ± 4.50
JinmiaoK2	K. Zhao	97.73 ± 2.32	1.79 ± 1.34
Datong29	K. Zhao	98.21 ± 1.62	16.33 ± 2.68
ShanxiHonggu	K. Zhao	96.79 ± 2.89	55.39 ± 6.85
Changnong47	K. Zhao	96.90 ± 2.73	43.27 ± 3.37
Longgu13	K. Zhao	96.92 ± 3.16	4.44 ± 1.41

Gonggu88	K. Zhao	95.81 ± 3.80	42.90 ± 3.27
Nenxuan18	K. Zhao	98.08 ± 1.91	43.35 ± 0.30
Zhangzagu13	K. Zhao	95.66 ± 4.41	0.71 ± 0.62
Longgu38	K. Zhao	96.58 ± 3.03	18.91 ± 3.20
Chaogu58	K. Zhao	98.06 ± 3.36	1.36 ± 0.59
HuangjinmiaoA	K. Zhao	97.09 ± 2.60	15.15 ± 2.19
Wansu66	J. Wang	97.52 ± 3.45	1.37 ± 0.60
Changsheng13	J. Wang	95.17 ± 0.73	0.71 ± 0.61
Jingu21Y	C.Y. Yang	88.37 ± 4.27	10.76 ± 2.48
Jingu40A	C.Y. Yang	37.46 ± 6.53	0.78 ± 1.34
Jingu29	C.Y. Yang	44.35 ± 9.94	0.00 ± 0.00
Miyou	J. Wang	91.21 ± 1.38	12.09 ± 3.99
Baguzi	J. Wang	85.65 ± 11.16	8.29 ± 2.20
Huangmaogu	J. Wang	97.33 ± 2.88	7.59 ± 1.70
HuangjinmiaoB	J. Wang	84.46 ± 5.60	0.00 ± 0.00
Baibazi	J. Wang	95.97 ± 4.13	10.12 ± 1.63
Fenxuan6	J. Wang	94.1 ± 6.01	0.00 ± 0.00
2003-384	J. Wang	72.71 ± 8.40	6.68 ± 1.23
HB12	J. Wang	97.93 ± 1.80	14.07 ± 4.05
Jinfen61	J. Wang	96.24 ± 2.76	16.31 ± 1.95
Se-kep-22	J. Wang	96.79 ± 3.30	0.00 ± 0.00
Jingu33	J. Wang	86.61 ± 8.03	3.59 ± 0.58
Dabaiagu	J. Wang	98.64 ± 1.52	1.75 ± 0.66
Jingu40B	J. Wang	83.47 ± 5.51	16.94 ± 0.99
Shandong-8	J. Wang	97.95 ± 2.06	10.04 ± 2.59
Fu1	J. Wang	78.23 ± 2.91	37.41 ± 1.45
Chao108	J. Wang	96.17 ± 5.78	33.75 ± 4.37
Shandong-5	J. Wang	90.03 ± 11.86	45.92 ± 3.23
Taixuan5	J. Wang	54.52 ± 9.26	41.18 ± 1.35
Zhang8311-13	J. Wang	98.64 ± 1.54	32.30 ± 3.88
Liuyuexian	J. Wang	97.43 ± 2.86	17.57 ± 2.61

Zhaonong40	J. Wang	55.25 ± 3.11	28.09 ± 7.67
Longzagu6	J. Wang	93.34 ± 9.76	9.70 ± 1.60
Jinpingu1	J. Wang	95.62 ± 3.79	70.22 ± 3.51
Changsui8	J. Wang	92.31 ± 4.51	48.73 ± 2.92
Jingu25	J. Wang	90.48 ± 5.19	58.73 ± 6.74
Jinfen17	J. Wang	97.94 ± 1.79	66.07 ± 2.27
Jiguang2	J. Wang	93.12 ± 3.04	25.56 ± 2.25
